# *Stimpsonia danxiacola* (Primulaceae), a new species endemic to danxia landscape from southern China

**DOI:** 10.3897/phytokeys.281.211660

**Published:** 2026-10-30

**Authors:** Xiao-Lan Zhao, Yan-Shuang Huang, Li-Juan Liao, Ling-Feng Peng, Qiang Fan

**Affiliations:** 1 State Key Laboratory of Biocontrol and Guangdong Provincial Key Laboratory of Plant Stress Biology, School of Life Sciences, Sun Yat-sen University, Guangzhou 510275, Chin State Key Laboratory of Biocontrol and Guangdong Provincial Key Laboratory of Plant Stress Biology, School of Life Sciences, Sun Yat-sen University Guangzhou China; 2 National Park and Nature Education Research Institute, Sun Yat-sen University, Guangzhou 510275, China National Park and Nature Education Research Institute, Sun Yat-sen University Guangzhou China; 3 Guangdong Danxiashan National Nature Reserve Administration, Shaoguan 512300, China Guangdong Danxiashan National Nature Reserve Administration Shaoguan China

**Keywords:** Danxia landscape, new species, phylogeny, Primulaceae, *
Stimpsonia
*

## Abstract

*Stimpsonia danxiacola* Xiao L.Zhao, Yan S.Huang & Q.Fan, sp. nov., a new species from southern China, is described and illustrated here, representing the third recognized species of the genus. Both morphological comparisons and phylogenetic analyses support *S. danxiacola* as a distinct species. The new species differs from *S. chamaedryoides* Wright ex A. Gray and *S. nanlingensis* G.H.Huang & G.Hao in its perennial habit, larger plant size, densely glandular-pubescent whole plant, viscid leaves, compound-racemiform inflorescences and calyx lobes which are reflexed at anthesis and gradually becoming connate in fruit, slightly exceeding the fruit.

## Introduction

*Stimpsonia* is a small genus of Primulaceae comprising only two currently recognized species worldwide (Huang and Hao 2017). The type species, *Stimpsonia chamaedryoides* Wright ex A.Gray, was first described in 1859 and is distributed in Japan and central China, including Guangxi, Guangdong, Hunan, Jiangxi, Anhui, Jiangsu, Zhejiang, Fujian, Guizhou, and Taiwan (Global Biodiversity Information Facility, GBIF, https://www.gbif.org/taxon/52QMG). This species commonly occurs in warm and humid environments, such as hills, low-mountain grasslands, and sparse forests. Huang and Hao (2017) described *S. nanlingensis* G.H.Huang & G.Hao as the second species, which is endemic to the Nanling Mountains.

The phylogenetic placement of *Stimpsonia*, known as the “*Stimpsonia problem*,” has long been controversial in Primulaceae s.l. Morphologically, it was traditionally placed in Primuloideae near *Androsace* based on shared floral traits (Anderberg et al. 2001). However, plastid phylogenetic analyses using *atpB*, *ndhF*, and *rbcL* placed *Stimpsonia* within Myrsinoideae rather than Primuloideae. Nuclear data later supported this placement, resolving *Stimpsonia* within the herbaceous clade of Myrsinoideae (Yan et al. 2025).

In December 2025, an unusual *Stimpsonia* species was discovered on Mount Bajiaozhai, a danxia landscape site in Ziyuan County, Guangxi, China. Living specimens with flower buds were collected during subsequent field investigations in January and April 2026. The plants were observed flowering from April to October, with fruiting starting before mid-May. Subsequent morphological comparisons and phylogenetic analyses finally support that it is a new species.

## Materials and methods

### Morphological examination

Specimens of the putative new species were collected from Mount Bajiaozhai in Ziyuan County, Guangxi, covering both flowering and fruiting stages. For comparative morphological analysis, we also included materials from two close relatives, *Stimpsonia chamaedryoides* (Matoushan National Nature Reserve, Jiangxi) and *S. nanlingensis* (Nanling National Forest Park, Guangdong).

Macromorphological characters (e.g., plant height, leaf length and width, petiole length, pedicel length, inflorescence branching pattern, corolla diameter, corolla tube length, style length, capsule diameter) were measured. Small floral characters were measured with a digital caliper to a precision of 0.1 mm, while gross morphological traits were measured with a scale ruler with a color chart to a precision of 1 mm. Micromorphological characters (e.g., glandular pubescence, corolla lobe shape, seed surface ornamentation) were observed and measured on specimens under a stereomicroscope fitted with an ocular micrometer, and photographed using a Sony α6000 camera equipped with a 100 mm macro lens (1:2.8 reproduction ratio). A millimeter scale bar was placed adjacent to each specimen during photography.

The protologues of *S. chamaedryoides* and *S. nanlingensis* were carefully consulted. Digital images of their type specimens were examined via the Chinese Virtual Herbarium (CVH; [https://www.cvh.ac.cn/] (https://www.cvh.ac.cn/)). Morphological accounts of both taxa from the Flora of China were also reviewed to support comprehensive morphological comparison. All voucher specimens are deposited in the Herbarium of Sun Yat-sen University (SYS).

### Molecular analyses

Fresh leaves were collected from three individuals of the putative new species, and from two individuals of *S. chamaedryoides*, and *S. nanlingensis*, respectively. Total genomic DNA was extracted from fresh leaves using the PlantZol reagent (catalog no. EE141-01; TransGen Biotech, Beijing, China) following the manufacturer’s instructions with minor modifications. The ITS region was amplified via polymerase chain reaction (PCR) using the primers ITS1 and ITS4 (White et al. 1990). The PCR amplifications were performed following Huang et al. (2021), sequenced using the Sanger method. A total of 14 accessions representing 12 Primulaceae species and two outgroup species were retrieved from GenBank (Zhang 2002; Schneeweiss et al. 2004; Kovtonyuk and Goncharov 2009; Casazza et al. 2012; Zhang et al. 2024) (Table 1). *Embelia floribunda* and *Heberdenia excelsa* were selected as outgroups (Kondraskov et al. 2015; Nongbet and Chrungoo 2018; Zhang et al. 2024). Including the newly generated ones, we used 21 sequences to reconstruct the phylogeny.

**Table 1.** GenBank accessions.

**Taxon**	**Locality**	**Reference**	**GenBank accessions**
*Primula lutea*	Austria: Portjoch	Huber et al., Unpublished	MW424635.1
*P. auricula*	/	Kovtonyuk and Goncharov 2009	AM920498.1
*P. albenensis*	Italy: Bergamo	Zhang 2002, Thesis	AJ427751.1
*P. allionii*	/	Casazza et al. 2012	JQ755782.1
*P. marginata*	/	Casazza et al. 2012	JQ755728.1
*P. apennina*	Italy: Apennines	Zhang 2002, Thesis	AJ427754.1
*P. kitaibeliana*	Croatia	Schneeweiss et al. 2004	AY275015.1
*P. wulfeniana*	Austria: Carinthia	Zhang 2002, Thesis	AJ427799.1
*P. spectabilis*	Italy: Mt Spino	Zhang 2002, Thesis	AJ427794.1
*P. clusiana*	Austria: Lower Austria	Zhang 2002, Thesis	AJ427770.1
*P. suffrutescens*	/	Kelso et al. 2008	EU887000.1
*P. parryi*	/	Kelso et al. 2008	EU886999.1
*Stimpsonia danxiacola*	China: Guangxi	Present Study	PZ804708
*S. danxiacola*	China: Guangxi	Present Study	PZ804709
*S. danxiacola*	China: Guangxi	Present Study	PZ804710
*S. nanlingensis*	China: Guangdong	Present Study	PZ804711
*S. nanlingensis*	China: Guangdong	Present Study	PZ804712
*S. chamaedryoides*	China: Jiangxi	Present Study	PZ804713
*S. chamaedryoides*	China: Jiangxi	Present Study	PZ804714
*Embelia floribunda*	/	Nongbet and Chrungoo 2018	MN689072.1
*Heberdenia excelsa*	Portugal: Madeira	Kondraskov et al. 2015	KJ189030.1

The Sanger sequencing chromatograms were examined using CHROMAS v2.6.6 (Technelysium Pty Ltd, South Brisbane, Australia), and sequences were manually trimmed and screened. We performed sequence alignment using MAFFT v7.525 (Katoh and Standley 2013) with the “--auto” option. Maximum likelihood (ML) phylogenetic inference was conducted using IQ-TREE v2.1.4 (Minh et al. 2020) under the TIM2+F+G4 substitution model with 2,000 ultrafast bootstrap replicates. Finally, we visualized and annotated the resulting tree using FIGTREE v1.4.4 (https://github.com/rambaut/figtree/releases).

## Results

Morphological comparisons revealed that *Stimpsonia danxiacola* differs from its congeners by a combination of diagnostic characters (Table 2). In the ITS-based ML phylogenetic tree, it was recovered as a distinct and strongly supported lineage (BS = 94%). Within the genus, *S. chamaedryoides* (BS = 98%) and *S. nanlingensis* (BS = 97%) formed a sister clade (BS = 90%), which is sister to *S. danxiacola*. The entire *Stimpsonia* clade receives full bootstrap support (BS = 100%).

**Table 2.** Morphological comparison among *Stimpsonia danxiacola*, *S. nanlingensis*, and *S. chamaedryoides*.

**Characte**rs	***Stimpsonia danxiacola***	***S. nanlingensis***	***S. chamaedryoides***
Cauline leaves	Cauline leaves gradually reduced toward the apex, becoming mostly bract-like, with a deeply cordate base.	Cauline leaves slightly reduced toward the apex, remaining entirely leaf-like, ovate to suborbicular.	Cauline leaves gradually reduced toward the apex, becoming mostly bract-like, lanceolate to linear.
calyx lobes	retroflexed when flowering, gradually coalesce in fruit	obviously retroflexed when flowering, spreading in fruit	scarcely retroflexed when flowering, spreading in fruit
corolla tube (mm)	ca 3.0	ca 1.5	ca 2.5
corolla lobe shape	oblong to obovate	oblong to obovate	cuneate-obovate
corolla lobe apex	deeply emarginate, sometimes irregularly undulate	truncate to obtuse, sometimes irregularly undulate	emarginate, sometimes undulate
Inflorescence	compound-racemiform inflorescence, 13–27.6 cm long	erect, simplly racemiform, ca 10 cm	erect, simplly racemiform, 8–10.7 cm long
height (cm)	22–27	3–13	ca 18
style (mm)	ca 1.2	shorter than 0.3	ca 0.6

### Taxonomic treatment

#### Stimpsonia danxiacola

Taxon classificationPlantaeEricalesPrimulaceae

Xiao L.Zhao, Yan S.Huang & Q.Fan sp. nov.

357A303A-7696-5EB1-B186-A74D085ED49D

urn:lsid:ipni.org:names:77484227-1

Figs 1, 2, 3, 4, 5

##### Type.

China • Preserved from a flowering plant cultivated at Sun Yat-sen University, originally collected in Guangxi Zhuang Autonomous Region, Guilin City, Ziyuan County, Mount Bajiaozhai, along a mountain roadside, 26.2946, 110.7068, 583 m a.s.l., 20 January 2026, *Li-Juan Liao & Xiao-Lan Zhao GX 24*; • flowering material preserved on 7 May 2026, *Xiao-Lan Zhao SD001* (holotype: SYS [SYS00237147]!).

**Figure 1.**
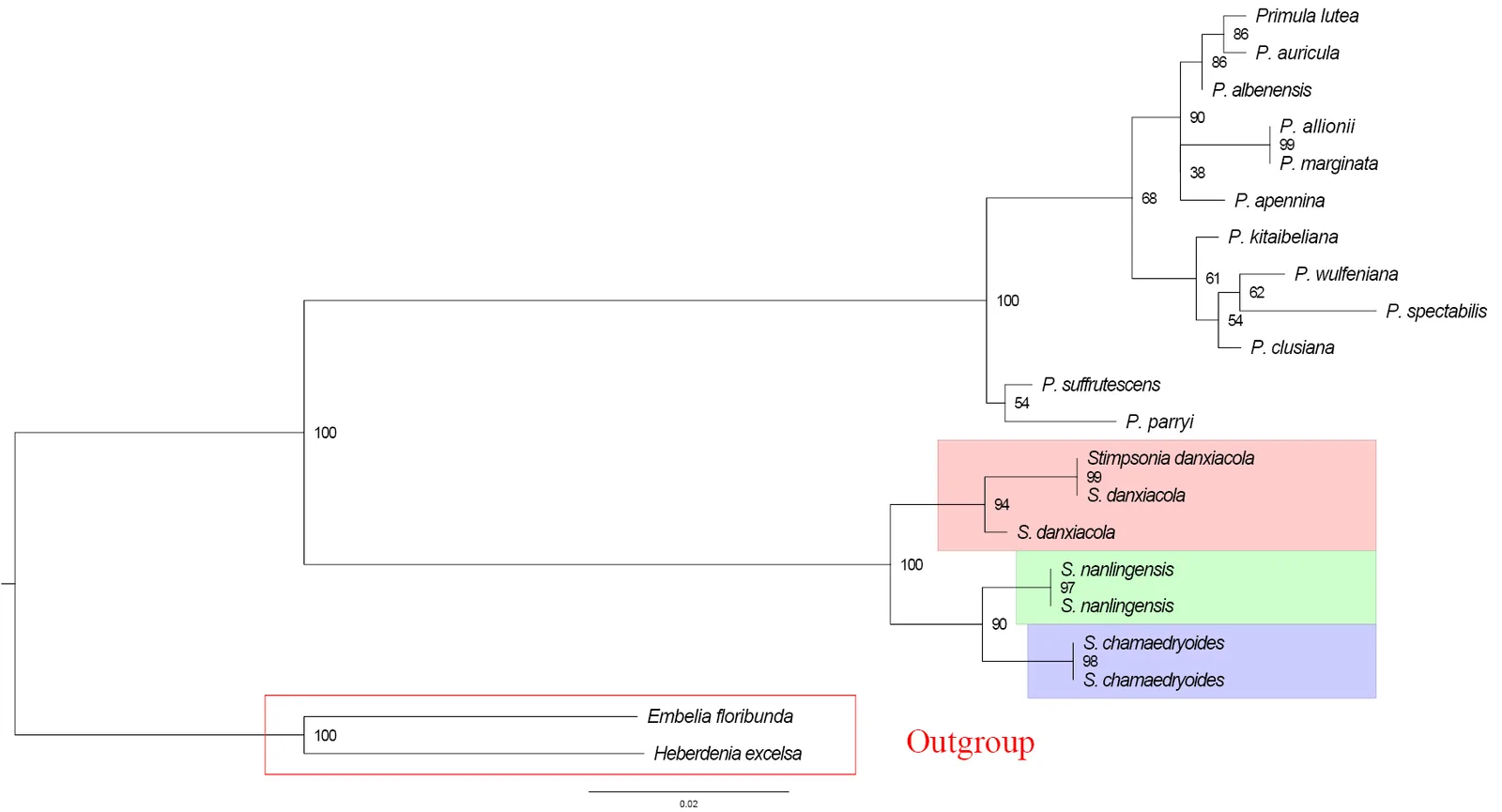
Maximum Likelihood phylogenetic tree constructed based on ITS sequences of *Stimpsonia danxiacola* and closely related species. Node support values are given as bootstrap support value (BS). *S. danxiacola* is highlighted in red, *S. nanlingensis* in green, and *S. chamaedryoides* in purple. Two outgroups are marked with red borders and annotated with red font alongside.

**Figure 2.**
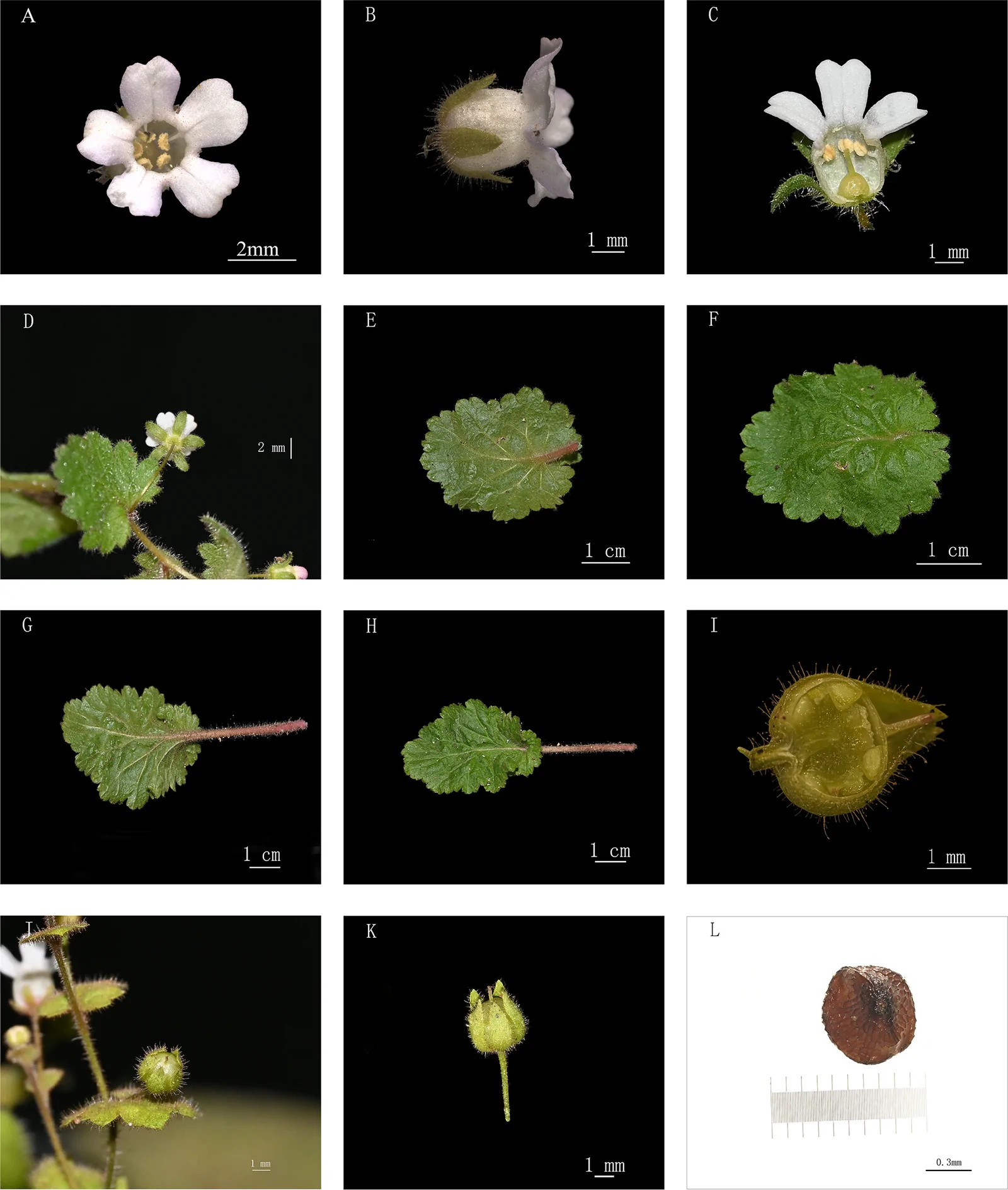
*Stimpsonia danxiacola*. **A**. Flower, aerial view; **B**. Flower, lateral view; **C**. Longitudinal section of flower; **D**. Flower in dorsal view showing calyx lobes; **E**. Abaxial view of a cauline leaf; **F**. Adaxial view of a cauline leaf; **G**. Abaxial view of a basal leaf; **H**. Adaxial view of a basal leaf; **I**. Longitudinal section of fruit; **J**. Fruit, aerial view; **K**. Fruit, lateral view; **L**. Seed. Photos by Xiao-Lan Zhao, Yan-Shuang Huang, Li-Juan Liao.

**Figure 3.**
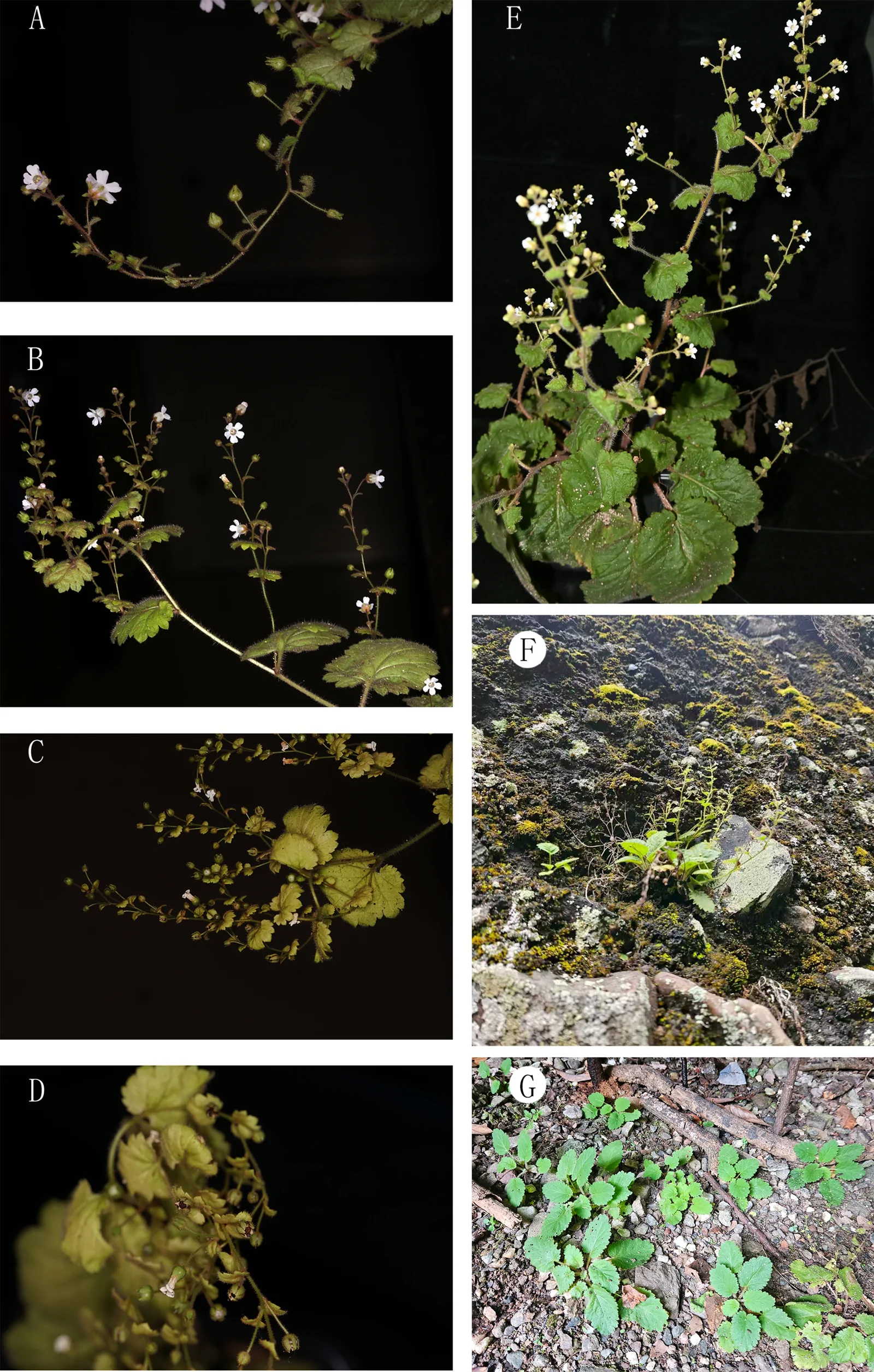
*Stimpsonia danxiacola*. **A**. Unbranched infructescence; **B**. Branched infructescence; **C, D**. Infructescence at seed maturity; **E**. Habit; **F**. Habitat; **G**. Young Plants.

**Figure 4.**
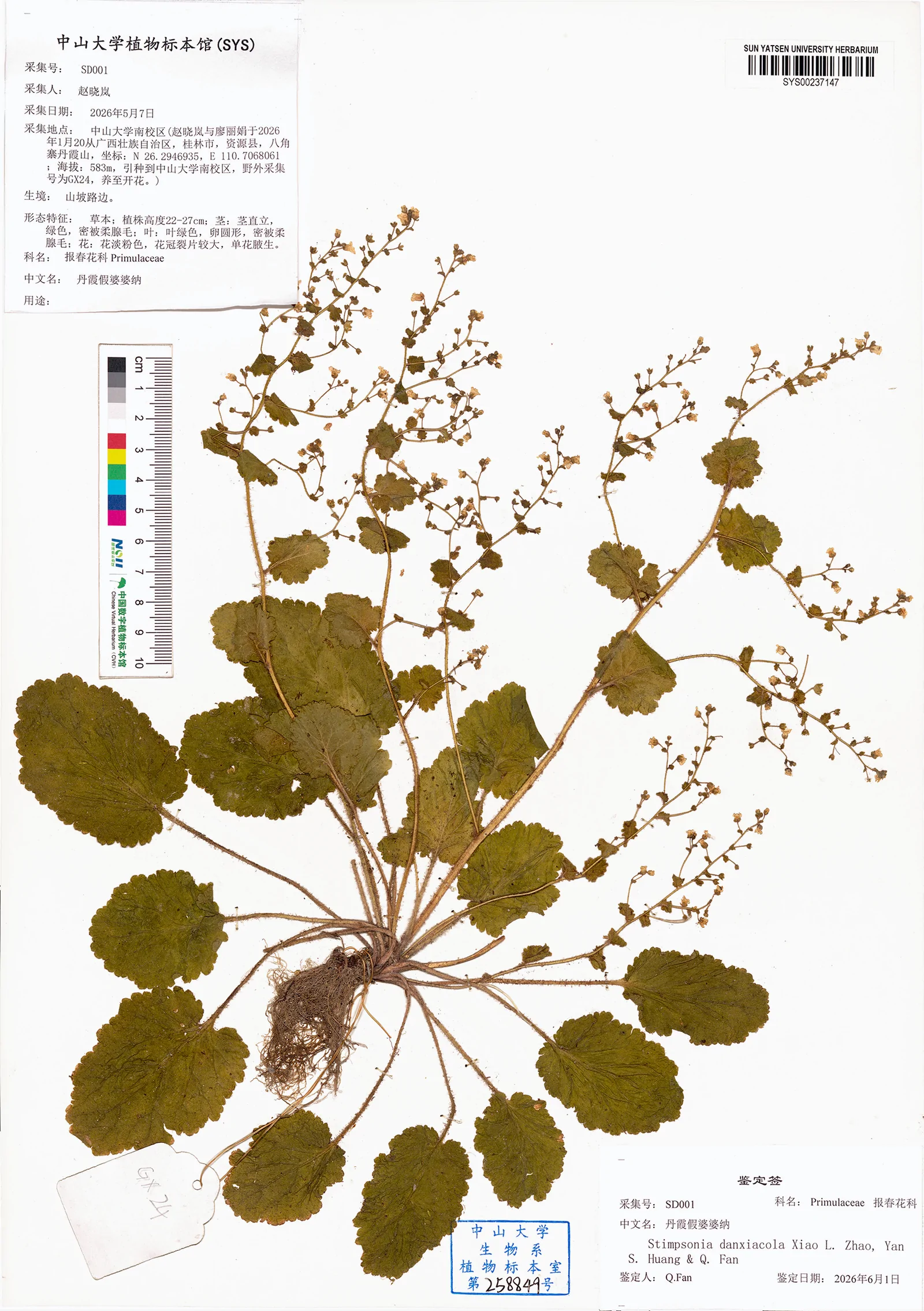
Holotype of *Stimpsonia danxiacola* sp. nov.

**Figure 5.**
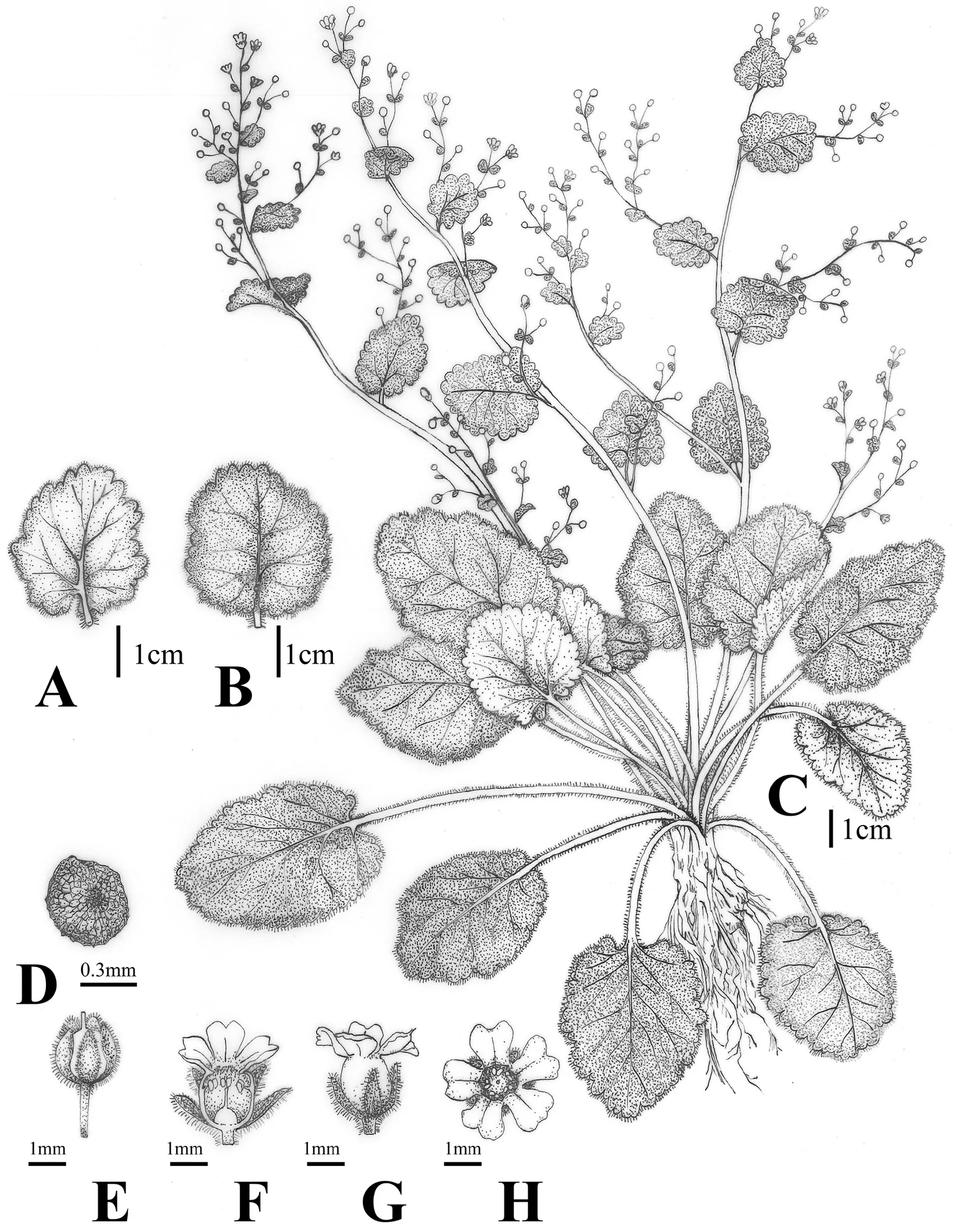
*Stimpsonia danxiacola*. **A**. Cauline leaf, abaxial view; **B**. Cauline leaf, adaxial view; **C**. Habit; **D**. Seed; **E**. Fruit with persistent connate calyx; **F**. Longitudinal section of flower; **G**. Lateral view of flower; **H**. Apical view of flower. Illustrated byYuan-Yuan Sun.

##### Diagnosis.

The new species differs from *Stimpsonia chamaedryoides* and *S. nanlingensis* in its perennial habit (vs. annual), densely glandular-pubescent plants with viscid leaves, compound racemiform inflorescences (vs. simple racemiform), long pedicels (0.4–2.6 cm vs. 2.0–8.0 mm and ca. 0.3 mm, respectively), pale pink flowers (vs. white and white or pink), and longer style (ca. 1.2 mm vs. ca. 0.6 mm and 0.3 mm, respectively). It further differs from *S. nanlingensis* in having calyx lobes that become connivent in fruit (vs. reflexed or patent).

##### Description.

Perennial herbs, marked individuals can resprout in successive years in the wild, 22–27 cm tall, densely glandular-pubescent throughout. Stem erect, solitary, occasionally branched, densely glandular-pubescent. Basal leaves petiolate; petioles 2.0–8.5 cm long, densely glandular-pubescent; blades elliptic to broadly ovate, 2.8–6.0 × 2.1–4.6 cm, green, chartaceous, with deeply cordate bases, densely glandular-pubescent on both surfaces, margins irregularly dentate. Cauline leaves alternate, 0.2–3.0 × 0.2–2.8 cm, densely glandular-pubescent on both surfaces; petioles gradually shortened upward; blades orbicular, with deeply cordate bases, dentate margins, and gradually reduced upward. Flowers axillary, solitary, arranged in a lax, spreading compound-racemiform inflorescence 13–27.6 cm long; primary branches 6.2–15 cm long, secondary branches 1.2–3.9 cm long; whole inflorescence densely glandular-pubescent; pedicels 0.4–2.6 cm long, densely glandular-pubescent. Calyx ca. 2.1 mm long, densely glandular-pubescent, 5-lobed nearly to the base; lobes reflexed at anthesis, gradually connivent in fruit. Corolla pale pink, ca. 4.8 mm long, 5.0–7.0 mm in diameter; tube ca. 3.0 mm long; lobes 5, obovate, 1.5–2 × 1.6–3 mm, apex emarginate, sometimes irregularly undulate, internally pubescent. Stamens ca. 1 mm long, labrous; anthers basifixed, subovoid, ca. 0.2 mm long, introrse. Ovary superior, ovoid, glabrous. Style ca. 1.2 mm long, persistent and exserted in fruit; stigma capitate. Capsule globose, ca. 3.0 mm in diameter, densely glandular-pubescent, shorter than the persistent calyx, dehiscent along dorsal sutures, valves spreading outwards. Seeds ca. 16 per capsule, depressed-globose, ca. 0.6 mm diam., brownish; testa conspicuously reticulate-foveolate with irregular lumina and slightly raised muri, and with a dark depressed area and a minute protrusion at the center.

##### Phenology.

Flowering from April to October; fruiting from May to October.

##### Etymology.

The specific epithet refers to the danxia landscape. The Chinese name is “丹霞假婆婆纳 (dān xiá jiă pó pó nà)”.

##### Distribution and habitat.

**Figure 6.**
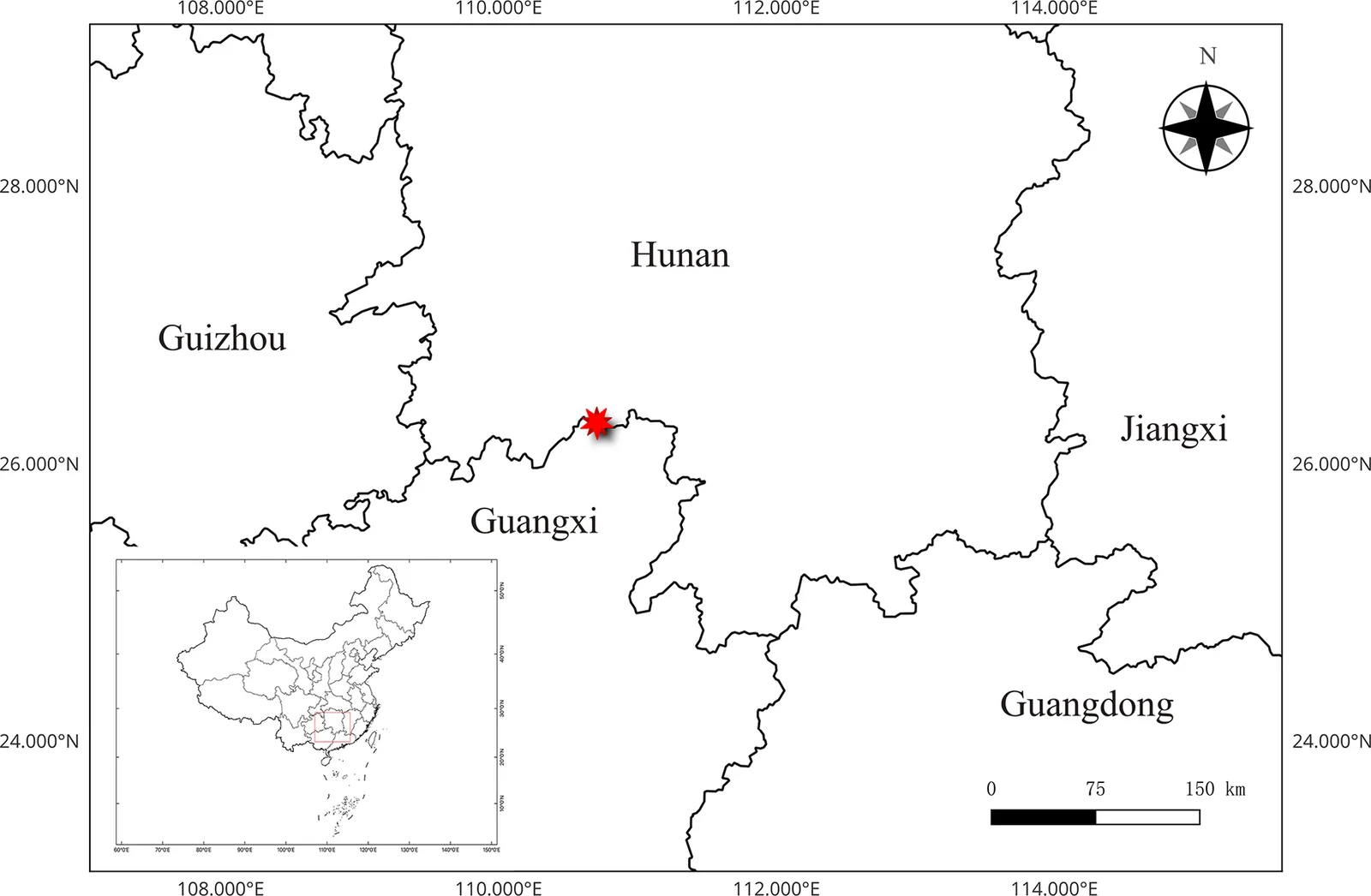
Distribution map of *Stimpsonia danxiacola*.

*S. danxiacola* is currently known from the danxia landscape of Ziyuan County, Guilin City, Guangxi (Fig. 6). Putative additional populations of this species occur in the adjacent Mount Langshan area of Hunan Province, as inferred from specimen records in the Chinese Virtual Herbarium (https://www.cvh.ac.cn/spms/detail.php?id=eee5d835). It grows along mountain roadsides in moist soil at elevations of 300–650 m.

##### Provisional conservation status.

The type locality lies near a hiking trail in the Danxia Scenic Area, which is subject to heavy tourism pressure and anthropogenic disturbance. This species is currently known from only a single confirmed locality: Bajiaozhai, Ziyuan County, Guilin City, Guangxi Zhuang Autonomous Region, at an elevation of 583 m, with approximately 40 mature individuals at this site. However, the full distribution range, population size, and habitat preference of this species remain poorly investigated. Considering that it may potentially occur in other underexplored danxia landscape habitats across the region, the currently available data is insufficient to accurately evaluate its conservation status. Following the IUCN Red List Categories and Criteria (IUCN 2001, version 3.1), we therefore propose a provisional conservation classification of Data Deficient (DD) until its actual distribution and population status are comprehensively clarified through further field investigations.

##### Additional specimens examined.

*Stimpsonia danxiacola*: China • Cultivated at Sun Yat-sen University, from living material originally collected in Guangxi Zhuang Autonomous Region, Guilin City, Ziyuan County, Mount Bajiaozhai, along a mountain roadside, 1 January 2026, *Li-Juan Liao & Xiao-Lan Zhao GX 25*; • fruiting material gathered on 26 May 2026, *Xiao-Lan Zhao SD002* (SYS [SYS00237148!, SYS00237149!]). • Hunan: Xinning County, Mount Langshan, under rocks, 300 m a.s.l., *Y.B. Luo 2279* (PE).

*Stimpsonia chamaedryoides*: China • Zhejiang Province: Nanping City, Pucheng County, Zhongxin Town, within Jiulongshan National Nature Reserve, alt. 888.39 m, 13 April 2018, *Xin Zhong, Shang Qu, Shu-Bo Huang ZX01647* (CSH); • Jiangxi Province: Zixi County, Matoushan Town, Zhuyan Mountain, 27°48'49"N, 117°13'27"E, 17 Oct 2025, *Yan-Shuang Huang JX16* (SYS!); • Zhejiang Province: Baishanzu, in valley forest, 16 April 2013, *Fang-Gang Zhang BSZ 0128* (ZMNH); • Jiangsu Province: Changzhou City, Liyang City, Songling of Liyang, 27 Apr 2007, *Q.X. Liu et al., LS-072*, (NAS, NAS00579254); • Fujian Province: Nanping City, Da'anyuan of Wuyi Mountain, roadside, 21 Jun 2017, *S.Z. Liu 3975*, (XMBG, XMBG0010427).

*Stimpsonia nanlingensis*: China • Guangdong Province: Ruyuan Yao Autonomous County, Nanling National Forest Park, 24°55'N, 113°03'E, 650 m a.s.l., 8 May 2016, *G.H. Huang 160033* (IBSC!); • Guangdong Province: Ruyuan Yao Autonomous County, Nanling National Forest Park, 24°57'N, 113°03'E, 1153 m a.s.l., 2 Jan 2026, *Qiang Fan, RY23* (SYS!).

## Discussion

Both the morphological comparisons and ITS-based phylogenetic analyses support that *S. danxiacola* is a distinct new species. The species possesses a stable combination of diagnostic characters, including perennial habit, larger plant size, dense glandular pubescence, viscid leaf surfaces, a compound-racemiform inflorescence, and distinct calyx characteristics, which clearly separate it from *S. chamaedryoides* and *S. nanlingensis* (Table 2). The ITS phylogeny recovered *S. danxiacola* as an independent lineage with strong bootstrap support (BS = 94%), providing additional support for its recognition.

## Supplementary Material

XML Treatment for Stimpsonia danxiacola

## Additional information

### Artificial Intelligence (AI) use

The authors accept full responsibility for the content of the manuscript, including the disclosure of any use of AI.

Regarding the use of AI in the preparation of this manuscript, the authors declare the following: DeepSeek, GPT.

**Used for**: Language, style and writing.

AI was used only for language editing and polishing of the manuscript. ChatGPT and DeepSeek were used to improve grammar, clarity, and academic English.

### Author ORCIDs

X.-L. Zhao https://orcid.org/0009-0004-7354-5922

L.-J. Liao https://orcid.org/0009-0008-3445-8809

Q. Fan https://orcid.org/0000-0003-4254-6936

### Data availability

All of the data that support the findings of this study are available in the main text.
